# Exotic complexes in one-dimensional Bose-Einstein condensates with spin-orbit coupling

**DOI:** 10.1038/s41598-018-22008-2

**Published:** 2018-02-27

**Authors:** D. Belobo Belobo, T. Meier

**Affiliations:** 1 African Center for Advanced Studies, P.O. Box, 4477 Yaounde, Cameroon; 2 PREPAVOGT Yaounde, P.O. Box 765 Yaounde, Cameroon; 3 Department of Physics and CeOPP, University of Paderborn, Warburger Strasse 100, D-33098 Paderborn, Germany

## Abstract

By means of the F-expansion method and intensive numerical simulations, the existence of three families of nonlinear matter waves including Jacobi elliptic functions, solitons, and triangular periodic functions, is demonstrated for spin-orbit coupled Bose-Einstein condensates with a linear potential. In addition, several complexes are obtained by taking two distinct solutions of each family or two distinct families. These solutions sustain different types of two-body interactions in the condensate that can be repulsive, attractive, or attractive and repulsive. Whereas the spin-orbit coupling destabilized these nonlinear matter waves, the linear potential leads to a stabilization. The numerical results are in excellent agreement with our analytical findings and it can be expected that the proposed robust solutions should be observable for experimentally relevant conditions.

## Introduction

Spin-orbit coupling (SOC) is an interaction between a quantum particle’s spin and its momentum^1,2^ which plays an important role in several areas in physics. SOC appears in condensed matter systems for example when electrons are placed in an electric or magnetic field, or possess strong SOC. Though neutral ultracold atomic systems do not have gauge coupling to electromagnetic fields nor SOC, in the few past years, the ability to control and manage the atom-light interaction in these settings allowed the creation of external Abelian or non-Abelian artificial gauge fields coupled to neutral atoms with many important implications^3^. Exploiting the possibility of creating gauge fields in ultracold atomics systems, in 2011, the group of Spielman first reported the observation of SOC in Bose-Einstein condensates (BECs)^4^. The latter work paved the way to the exploration of SOCs physics in ultracold neutral atomic gases for many reasons^5,6^. SOC is at the origin of important concepts in condensed matter systems such as the spin Hall effect and topological insulators^7–9^. It is also believed that SOC is also of relevance for the fundamental and exotic physics of superfluids, fermions and BECs^4,7–9^.

In recent years, many studies of BECs with SOC have revealed several interesting aspects like the partial wave scattering^10^, the phenomenon of Zitterbewegung (ZB)^11^, the tunability of the SOC strength^12^, the existence of a ‘stripe phase’^13,14^, vortices with^15^ or without^16,17^ rotation, tunneling dynamics^18–21^, and nonlinear matter waves^15–17,22–32^. Most of the above mentioned studies on nonlinear matter waves consider BECs with SOCs confined either in optical lattices^22–25^, or in harmonic potentials^26–29^, or self-trapped condensates^30–32^ focus on solitons, i.e., topological excitations of nonlinear systems with a broad range of applications^33^. BECs with SOCs loaded in a linear potential have not been investigated so far and neither periodic nonlinear waves nor the existence of complexes like a soliton with a periodic wave have been considered.

It has been shown that periodic waves can be found in single BECs with a linear potential^34–37^ and in self-trapped coupled BECs^38,39^. In this work, we report the existence and dynamics of ‘exotic’ complexes in BECs with SOC in the presence of a linear potential. These complexes consist of combinations of nonlinear matter waves such as solitons with Jacobi elliptic function (JEF), two JEF, or two triangular periodic function solutions, as well as bright-bright, dark-dark, and bright-dark solitons. The solutions to be constructed are stable in the absence of the linear potential provided that the strength of the SOC is sufficiently small, but they are unstable for large values of the SOC. However, the instability is wiped out when the linear potential is taken into account. The parameters used in our analytical and numerical studies are close to realistic experimental conditions and it can thus be expected that the predicted nonlinear matter waves may be observed in current experiments.

## Results

### Model

The nonlinear dynamics of BECs with SOC in quasi-one dimension is described by the coupled Gross-Pitaevskii equations in the mean-field limit^1,2,5,6^

1 $$\begin{array}{rcl}i\frac{\partial {\psi }_{j}}{\partial t} & = & \frac{-1}{2}\frac{{\partial }^{2}{\psi }_{j}}{\partial {x}^{2}}+i{(-\mathrm{1)}}^{j}\gamma \frac{\partial {\psi }_{j}}{\partial x}\\  &  & +\,[V(x)+{g}_{jj}|{\psi }_{j}{|}^{2}+{g}_{j3-j}|{\psi }_{3-j}{|}^{2}]{\psi }_{j},\quad j=1,2\end{array}$$

in which the linear cross coupling Rabi term $\frac{R}{2}{\psi }_{3-j}$ of strength *R* has been set to zero (*R* = 0) for simplicity^40^. Such a situation was recently suggested in ref.^40^ since the presence of the cross coupling Rabi term does not impact the stability of the condensates and merely induces small stripes for small values of *R*. In Eq. (1), space and time are measured in units of *ζ* = 1 *μm* and *mζ*^2^/*ħ*, respectively, *m* being the reduced mass, and *ħ* the Planck’s constant. *ψ*_*j*_ ( *j* = 1, 2) denotes the two pseudospin components of the condensate wave function. The term $\mp i\gamma \frac{\partial {\psi }_{j}}{\partial x}$ represents the momentum transfer between the laser beams and the atoms arising from the SOC while, *g*_*jj*_ and *g*_*j*3−*j*_ are the two-body intra and inter atomic interaction strengths, respectively. For simplicity, we consider here *g* = *g*_12_ = *g*_21_. *V*(*x*) = *βx* is the external linear potential that may mimic the gravitational field experienced by atoms due to their mass or an exposure of the condensate to a linear force realized by appropriate laser beams^34–37^. This may be explained by the fact that atoms of the condensates which are in the nK-mK regime have a nonzero mass such that the effect of the gravitational field is no longer negligible. It has been shown that the gravitational field explains the vortex fragmentation during topological phase imprinting observed in the Kyoto experiment unless the field time reverse belongs to a narrow window^41,42^. In condensate experiments, a linear potential may be realized by an exposure of the condensate to a laser beam with an appropriate wavelength^34–37^. Therefore, a linear potential acting along the free axis here *x* is the general form of the field which may be, in a specific case, represented by the gravitational field^34–37^. Very recently, Belobo showed that the linear potential stabilizes unstable bright solitons in a derivative Gross-Pitaevskii model of condensates^43^. Moreover, the linear potential is a key ingredient to explain the acceleration and dynamics of nonlinear waves in other media such as laser pulses in fiber optics, Langmuir waves in plasma physics, one-dimensional water channel gravity waves in hydrodynamics, extreme nonlinear waves with possible applications to optical soliton supercontinuum generation and ocean coast line protection^44^ and references therein. The set of Eq. (1) (and its variant forms) represents a quite general system of nonlinear evolution equations which also appears in other fields in physics such as field theory and the massive Thirring model, in optical fiber gratings, birefringent optical fibers, coupled optical wave guides and so on, see, e.g.^26–32^, and references therein.

### Analytical results

Here we adopt the F-expansion method^45^ in order to construct solutions of Eq. (1). We use the Ansatz

2 $${\psi }_{j}={h}_{j}{{\varphi }}_{j}(\xi )\,\exp \,[i{\theta }_{j}(x,t)],\quad j=1,2$$

where *ξ* = *k*(*t*)*x* + *n*(*t*) is the one phase-traveling variable and *θ*_*j*_(*x*, *t*) = Γ_*j*_(*t*)*x* + Ω_*j*_(*t*) and Γ_*j*_(*t*), Ω_*j*_(*t*) are the linear frequency shift and the homogeneous phase, respectively.

The function *ϕ*_*j*_(*ξ*) satisfies the auxiliary equation^46^

3 $$\frac{d{{\varphi }}_{j}(\xi )}{d\xi }={[{b}_{0j}+{b}_{2j}{{\varphi }}_{j}^{2}(\xi )+{b}_{4j}{{\varphi }}_{j}^{4}(\xi )]}^{\mathrm{1/2}},\quad \,j=1,2$$

with coefficients *b*_*mj*_, *m* = 0, 2, 4, being real constants. The solutions of Eq. (3) can be found in Table 1. Inserting Eq. (2) into Eq. (1) one obtains, after a little algebra, the following relations

4 $$k(t)=k,\quad \,{{\rm{\Gamma }}}_{1}(t)=-\beta t+{{\rm{\Gamma }}}_{10},\quad \,{{\rm{\Gamma }}}_{2}(t)={{\rm{\Gamma }}}_{1}+{{\rm{\Gamma }}}_{20},$$

5 $$n=k[\frac{\beta }{2}{t}^{2}-\frac{({{\rm{\Gamma }}}_{20}+{{\rm{\Gamma }}}_{10})t}{2}]+{n}_{0},\quad \,\gamma =\frac{{{\rm{\Gamma }}}_{20}-{{\rm{\Gamma }}}_{10}}{2},$$

6 $${b}_{4j}=\frac{{g}_{jj}{h}_{j}^{2}}{{k}^{2}},\quad \,{{\rm{\Omega }}}_{j}=\int [\frac{1}{2}({k}^{2}{b}_{2j}-{{\rm{\Gamma }}}_{j}^{2})+{(-\mathrm{1)}}^{j}\gamma {{\rm{\Gamma }}}_{j}]dt-g\int {h}_{3-1}^{2}{{\varphi }}_{3-j}^{2}dt,{{\varphi }}_{1}\ne {{\varphi }}_{2},$$

7 $${b}_{4j}=\frac{{g}_{jj}{h}_{j}^{2}+g{h}_{3-j}^{2}}{{k}^{2}},\quad {{\rm{\Omega }}}_{j}=\int [\frac{1}{2}({k}^{2}{b}_{2j}-{{\rm{\Gamma }}}_{j}^{2})+{(-\mathrm{1)}}^{j}\gamma {{\rm{\Gamma }}}_{j}]dt,\quad {{\varphi }}_{1}={{\varphi }}_{2},$$

*h*_*j*_, *g*_*jj*_

**Table 1:** Classification of different solutions. 0 ≤ *K* ≤ 1 is the modulus of the JEF, ${K}_{1}=\sqrt{1-{K}^{2}}$, *D*_1_, *D*_2_, *D*_3_(*D*_1_*D*_2_*D*_3_ ≠ 0) are arbitrary real constants.

Family	p	*ϕ*(*ξ*)	Conditions
JEF	1	*cd*(*ξ*), *sn*(*ξ*)	*b*_0_ = 1, *b*_2_ = −(1 + *K*^2^), *b*_4_ = *K*^2^
	2	*ns*(*ξ*), *dc*(*ξ*)	*b*_0_ = *K*^2^, *b*_2_ = −(1 + *K*^2^), *b*_4_ = 1
	3	*dn*(*ξ*)	*b*_0_ = *K*^2^ − 1, *b*_2_ = 2 − *K*^2^, *b*_4_ = −1
	4	*cn*(*ξ*)	*b*_0_ = 1 − *K*^2^, *b*_2_ = 2*K*^2^ − 1, *b*_4_ = −*K*^2^
	5	*nc*(*ξ*)	*b*_0_ = −*K*^2^, *b*_2_ = −1 + 2*K*^2^, *b*_4_ = 1 − *K*^2^
	6	*nd*(*ξ*)	*b*_0_ = −1, *b*_2_ = 2 − *K*^2^, *b*_4_ = *K*^2^ − 1
	7	*cs*(*ξ*)	*b*_0_ = 1 − *K*^2^, *b*_2_ = 2 − *K*^2^, *b*_4_ = 1
	8	*sc*(*ξ*)	*b*_0_ = 1, *b*_2_ = 2 − *K*^2^, *b*_4_ = 1 − *K*^2^
	9	*sd*(*ξ*)	*b*_0_ = 1, *b*_2_ = 2*K*^2^ − 1, *b*_4_ = *K*^2^(−1 + *K*^2^)
	10	*ds*(*ξ*)	*b*_0_ = *K*^2^(−1 + *K*^2^), *b*_2_ = 2*K*^2^ − 1, *b*_4_ = (1 − *K*^2^)/4
	11	*ns*(*ξ*) ± *cs*(*ξ*)	*b*_0_ = 1/4, *b*_2_ = (1 − 2*K*^2^)/2, *b*_4_ = 1/4
	12	*nc*(*ξ*) ± *sc*(*ξ*)	*b*_0_ = (1 − *K*^2^)/4, *b*_2_ = (1 + *K*^2^)/2, *b*_4_ = (1 − *K*^2^)/4
	13	*ns*(*ξ*) + *ds*(*ξ*)	*b*_0_ = *K*^2^/4, *b*_2_ = (*K*^2^ − 2)/2, *b*_4_ = 1/4
	14	*sn*(*ξ*)*dn*(*ξ*)/*cn*(*ξ*)	*b*_0_ = 1, *b*_2_ = 2 − 4*K*^2^, *b*_4_ = 1
	15	*dn*(*ξ*)*cn*(*ξ*)/*D*_1_[1 + *sn*(*ξ*)][1 + *Ksn*(*ξ*)]	${b}_{0}=(K-{\mathrm{1)}}^{2}\mathrm{/4}{D}_{1}^{2}$, *b*_2_ = (1 + *K*^2^ + 6*K*)/2, ${b}_{4}={D}_{1}^{2}{(-1+K)}^{2}\mathrm{/4}$
	16	*dn*(*ξ*)*cn*(*ξ*)/*D*_1_[1 + *sn*(*ξ*)][1 − *Ksn*(*ξ*)]	*b*_0_ = (*K* + 1)^2^/4*D*^2^1, *b*_2_ = (1 + *K*^2^ − 6*K*)/2, ${b}_{4}={D}_{1}^{2}\mathrm{(1}+{K}^{2}\mathrm{)/4}$
	17	*Kdn*(*ξ*)*cn*(*ξ*)/[1 + *Ksn*^2^(*ξ*)]	*b*_0_ = −2*K*^3^ + *K*^4^ + *K*^2^, *b*_2_ = 6*K* − *K*^2^ − 1, b = −4/*K*
	18	*Kdn*(*ξ*)*cn*(*ξ*)/[−1 + *Ksn*^2^(*ξ*)]	*b*_0_ = 2*K*^3^ + *K*^4^ + *K*^2^, *b*_2_ = −6*K* − *K*^2^ − 1, b = 4/*K*
	19	*K*^2^*sn*(*ξ*)*cn*(*ξ*)/[*K*_1_ − *dn*^2^(*ξ*)]	*b*_0_ = 2 + 2*K*_1_ − *K*^2^, *b*_2_ = 6*K*1 − *K*^2^ + 2, b = 4*K*_1_
	20	−*K*^2^*sn*(*ξ*)*cn*(*ξ*)/(*K*_1_ + *dn*^2^(*ξ*))	*b*_0_ = 2 − 2*K*_1_ − *K*^2^, *b*_2_ = −6*K*1 − *K*^2^ + 2 b_4_ = −4*K*_1_
	21	$\frac{\sqrt{\frac{{D}_{2}^{2}-{D}_{3}^{2}}{{D}_{2}^{2}-{D}_{3}^{2}{K}^{2}}}+sn(\xi )}{{D}_{2}cn(\xi )+{D}_{3}dn(\xi )}$	${b}_{0}=({K}^{2}-\mathrm{1)/4(}{D}_{3}^{2}{K}^{2}-{D}_{2}^{2})$, *b*_2_ = (*K*^2^ + 1)/2, ${b}_{4}=({D}_{3}^{2}{K}^{2}-{D}_{2}^{2})({K}^{2}-\mathrm{1)/4}$
	22	$\frac{\sqrt{\frac{{D}_{2}^{2}+{D}_{3}^{2}-{D}_{3}^{2}{K}^{2}}{{D}_{2}^{2}+{D}_{3}^{2}}}+dn(\xi )}{{D}_{2}sn(\xi )+{D}_{3}cn(\xi )}$	${b}_{0}={K}^{4}\mathrm{/4(}{D}_{2}^{2}+{D}_{3}^{2})$, *b*_2_ = *K*^2^/2 − 1, ${b}_{4}=({D}_{2}^{2}+{D}_{3}^{2}\mathrm{)/4}$
	23	[*Ksn*^2^(*ξ*) − 1]/*D*_2_[*Ksn*^2^(*ξ*) + 1]	${b}_{0}\mathrm{=(2}K-{K}^{2}-\mathrm{1)/}{D}_{2}^{2}$, *b*_2_ = 2*K*^2^ + 2, ${b}_{4}=-{D}_{2}^{2}({K}^{2}+1+2K)$
	24	[*Ksn*^2^(*ξ*) + 1]/*D*_2_[*Ksn*^2^(*ξ*) − 1]	${b}_{0}=-\mathrm{(2}K+{K}^{2}+\mathrm{1)/}{D}_{2}^{2}$, *b*_2_ = 2*K*^2^ + 2, ${b}_{4}=-{D}_{2}^{2}({K}^{2}+1+2K)$
	25	*Kns*(*ξ*) ± *cs*(*ξ*), *sn*(*ξ*)/[1 ± *cn*(*ξ*)], *cn*(*ξ*)/[(1 − *K*^2^)^1/2^*sn*(*ξ*) ± *dn*(*ξ*)]	*b*_0_ = *b*_4_ = 1/4, *b*_2_ = (1 − 2*K*^2^)/2
	26	*dn*(*ξ*)/[1 ± *Ksn*(*ξ*)], *Ksd*(*ξ*) ± *nd*(*ξ*)	*b*_0_ = *b*_4_ = (*K*^2^ − 1)/4, *b*_2_ = (*K*^2^ + 1)/2
	27	*cn*(*ξ*)/[1 ± *sn*(*ξ*)], *nc*(*ξ*) ± *sc*(*ξ*)	*b*_0_ = *b*_4_ = (1 − *K*^2^)/4, *b*_2_ = (*K*^2^ + 1)/2
	28	*Kcn*(*ξ*) ± *dn*(*ξ*)	*b*_0_ = −(1 − *K*^2^)^2^/4, *b*_2_ = (*K*^2^ + 1)/2, *b*_4_ = −1/4
	29	*sn*(*ξ*)/*dn*(*ξ*) ± *cn*(*ξ*)	*b*_0_ = 1/4, *b*_2_ = (*K*^2^ + 1)/2, *b*_4_ = (1 − *K*^2^)^2^/4
	30	$cn(\xi )/[\sqrt{1-{K}^{2}}\pm dn(\xi )],sn(\xi \mathrm{)/[1}\pm dn(\xi )]$	*b*_0_ = 1/4, *b*_2_ = (*K*^2^ − 2)/2, *b*_4_ = *K*^4^/4
Solitons	31	$\sqrt{-{b}_{2}/{b}_{4}}sech(\sqrt{{b}_{2}}\xi )$	*b*_0_ = 0, b_2_ > 0, *b*_4_ < 0
	32	$\sqrt{{b}_{2}/{b}_{4}}csch(\sqrt{{b}_{2}}\xi )$	*b*_0_ = 0, b_0_ => 0, *b*_4_ > 0
	33	$\sqrt{-{b}_{2}\mathrm{/2}{b}_{4}}{\rm{t}}{\rm{a}}{\rm{n}}{\rm{h}}(\sqrt{-{b}_{2}\mathrm{/2}}\xi )$	${b}_{0}={b}_{2}^{2}\mathrm{/4}{b}_{4}$, b_2_ < 0, b_4_ > 0
	34	$\pm \sqrt{2-2{{\rm{t}}{\rm{a}}{\rm{n}}{\rm{h}}}^{2}({D}_{4}-\xi )}/{\rm{t}}{\rm{a}}{\rm{n}}{\rm{h}}({D}_{4}-\xi )$	*b*_0_ = 0, b = 1, *b*_2_ = 1/2
Triangular periodic	35	$\sqrt{-{b}_{2}/{b}_{4}}{\rm{s}}{\rm{e}}{\rm{c}}(\sqrt{-{b}_{2}}\xi ),\sqrt{-{b}_{2}/{b}_{4}}{\rm{c}}{\rm{s}}{\rm{c}}(\sqrt{-{b}_{2}}\xi )$	${b}_{0}={b}_{2}^{2}\mathrm{/4}{b}_{4}$, b_2_ < 0, b_4_ > 0
	36	$\sqrt{-{b}_{2}\mathrm{/2}{b}_{4}}{\rm{t}}{\rm{a}}{\rm{n}}(\sqrt{{b}_{2}\mathrm{/2}}\xi )$	${b}_{0}={b}_{2}^{2}\mathrm{/4}{b}_{4}$, b_2_ > 0, b_4_ > 0

being free real constants.

All relevant information about the solutions of Eq. (1) can be deduced from Eqs (4–7). For example, Eq. (5) implies that the SOC strength must be half of the linear frequency shift difference at the initial time. In other words, the experiment needs to be performed such that the initial phase difference of the pseudospin components be twice the strength of the SOC parameter *γ*. Furthermore, Eqs (5–6) tell us that the values of two-body interactions of the condensate can be chosen at will, such that it is simply possible to fit them with those used in current experiments where *g*_11_ = *g*_22_ ≈ *g*^4–6^. One may also be interested in the dynamics of symbiotic BECs with SOC where the two-body interactions in the condensates might have opposite signs, i.e., *g*_11_*g*_22_ < 0. Such a situation is realizable in BECs due to the Feshbach resonance management technique^47^. In addition, the position, velocity, and acceleration of the center of mass of each pseudospin component is given by ${X}_{CM}=-[\frac{\beta }{2}{t}^{2}-\frac{({{\rm{\Gamma }}}_{20}+{{\rm{\Gamma }}}_{10})t}{2}]-\frac{{n}_{0}}{k}$, ${\dot{X}}_{CM}=-[\beta t-\frac{({{\rm{\Gamma }}}_{20}+{{\rm{\Gamma }}}_{10})}{2}]$, ${\ddot{X}}_{CM}=-\beta$, respectively. The mean dynamics of the pseudospin components depends mainly on the strength of the linear potential and the homogeneous phases at initial time. The linear potential might be used in BECs with SOC with applications to their transport or the realization of atomic spin-orbit BEC lasers, in a similar fashion as in single condensates^34–37^.

The functions *ϕ*_1,2_(*ξ*) dictate the profiles of the solutions. The explicit expressions of our solutions of Eq. (1) are *ψ*_*j*,*p*_ (*x*,*t*) = *h*_*j*_*ϕ*_*j*,*p*_ (*ξ*)exp[*iθ*_*j*_(*x*, *t*)] where *j* = 1, 2 and $p=\overline{1-36}$. There are three families of solutions provided in Table 1 which are JEFs ($p=\overline{1-30}$), solitons ($p=\overline{31-34}$), and triangular periodic solutions ($p=\overline{35-36}$). The integer *p* indicates the solution of Eq. (3) one chooses in Table 1. One should note that JEFs degenerate to other functions when *K* takes some special values. To be precise, *dn*(*ξ*, 0) = 1, *cn*(*ξ*, 0) = cos(*ξ*), *sn*(*ξ*, 0) = sin(*ξ*) and *dn*(*ξ*, 1) = *cn*(*ξ*, 1) = *sech*(*ξ*), *sn*(*ξ*, 1) = tanh(*ξ*) hereby enriching the number of solitons and triangular periodic solutions. For *ϕ*_1_(*ξ*) = *ϕ*_2_(*ξ*), families of periodic solutions in BECs with SOC like JEFs and triangular periodic solutions proposed here have not been reported yet. Moreover, novel exotic complexes are obtained for *ϕ*_1_(*ξ*) ≠ *ϕ*_2_(*ξ*). Such complexes include two mixed solutions of each family or two distinct families. These solutions thus open the route to explorations of new physical phenomena in BECs with SOC and related fields which is different to the well known solitons (bright-bright, bright-dark, dark-dark, see^22–32^) that are commonly studied in these media.

### Numerical results

We perform numerical simulations in order to test the robustness of the solutions that are constructed analytically as only robust or stable waves might be observable in real experiments. We consider a quasi-one-dimensional BEC with two-body intra and inter pseudospin interaction strengths being |*g*_*jj*_| = 1, and *g* = 0.8 and take the SOC’s strength in the interval 0 ≤ *γ* ≤ 1.5. Our numerical simulations run up to *t* = 1000 which corresponds to ≈1.342 s for ^87^Rb atoms with repulsive interactions^4^ (≈0.111 ms for ^7^Li atoms with attractive interactions^28^). The split-step Fourier method is used to integrate the set of Eq. (1). We have used 4096 points in the integration domain of length *x* ∈ [−1500, 1500] and the time step was dt = 0.001. A random amplitude-phase perturbation of one percent strength the maximum of the initial wave functions was used to launch the integration. In the following, we discuss and analyze the dynamics for some interesting profiles of solutions that could be realizable in experiments.

### Periodic solutions

**Figure 1:**
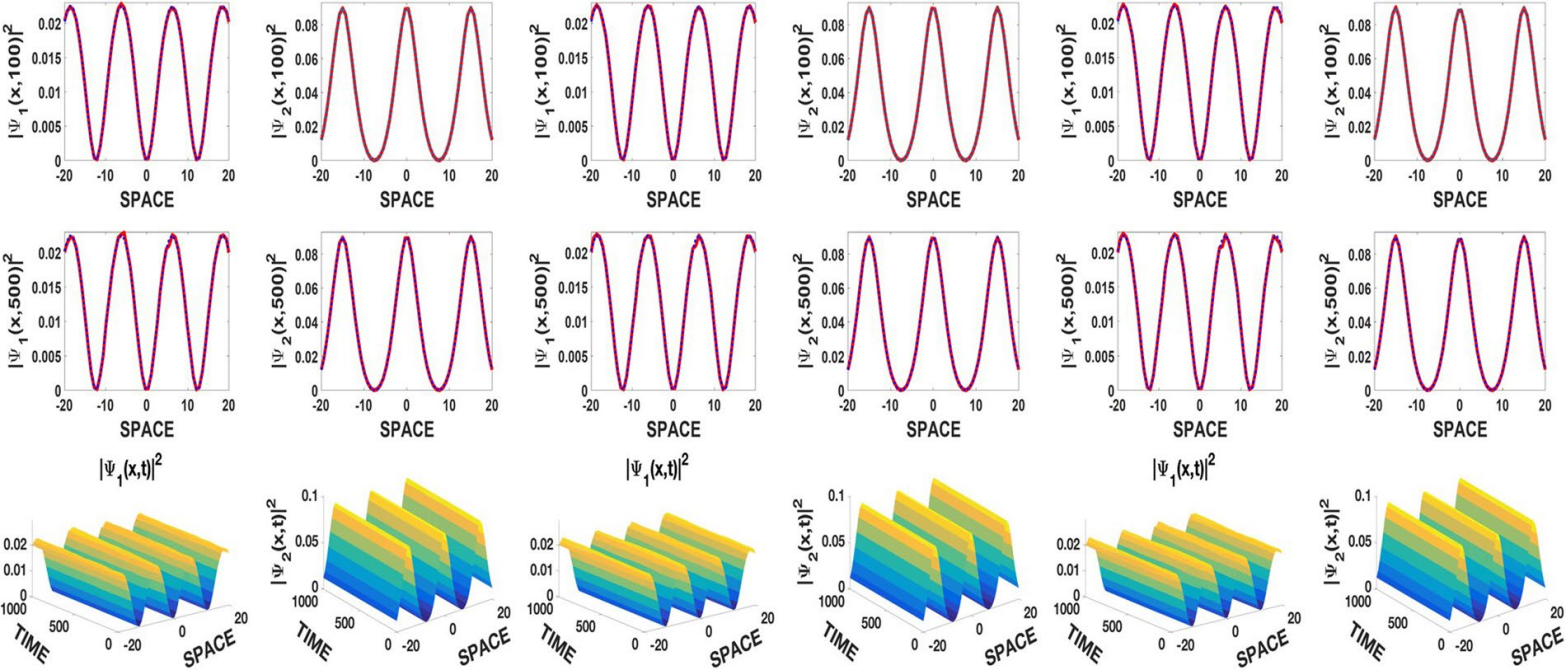
Top and middle rows: spatial comparison between numerical (red solid line) and analytical (blue dotted line) solutions at t = 100 and 500, respectively. Robust numerical solutions in bottom row. The columns are in pairs of |*ψ*_*j*_|^2^(*j* = 1, 2), left columns *g*_11_ = *g*_22_ = 1, middle columns |*ψ*_*j*_|^2^, *g*_11_ = *g*_22_ = −1, right columns |*ψ*_*j*_|^2^, *g*_11_ = −1, *g*_22_ = 1 and *g* = 0.8, *γ* = 0.01, *β* = 0, *p* = 1 with sn for *ψ*_1_, *p* = 3 for *ψ*_2_ everywhere. Other parameters are: *k* = 0.3, *k*_1_ = 0.5, *k*_2_ = 0.8, Γ_10_ = *π*, *n*_0_ = 0.

**Figure 2:**
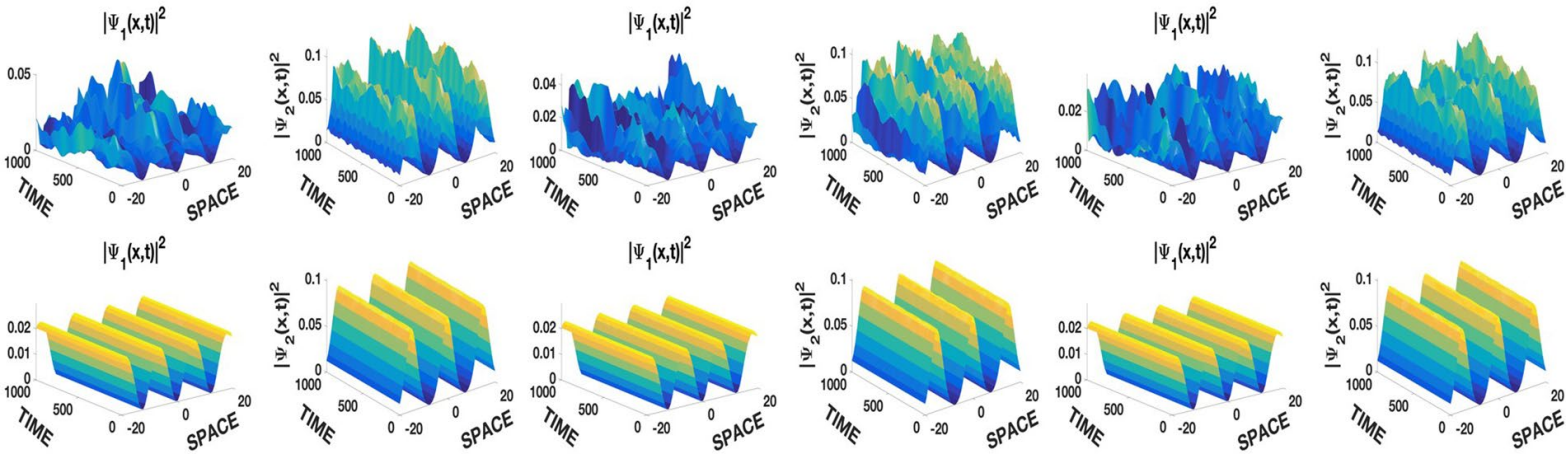
Spatiotemporal evolution of densities, *γ* = 0.5. Top row, *β* = 0, unstable self-trapped states. Bottom row, *β* = 0.1, stabilization of unstable solutions of the first row by the linear potential. All other conditions and parameters are the same as in Fig. 1.

Figure 1 displays robust JEF solutions of types sn and dn for the pseudospins *ψ*_1_ and *ψ*_2_ with repulsive, attractive, and symbiotic two-body interactions in the left, middle and, right columns, respectively. The SOC is *γ* = 0.01 while the external linear potential is turned off (*β* = 0). The first and second rows in Fig. 1 show a very good agreement between the analytical and the numerical solutions for times *t* = 100 and 500, respectively. The third row which exhibits the spatiotemporal evolution of the pseudospin densities moving at the constant velocity *V* = *γ* = 0.01 confirms their robustness due to their long time behavior without disintegration. However, not all values of the SOC parameter *γ* allow robust self-trapped solutions. Indeed, after intensive numerical simulations, we deduced that self-trapped solutions are stable for $0.015\,\lessapprox \,\gamma$. In other words, SOC strengths *γ* above the critical value *γ*_*crit*_ ≈ 0.015 destabilize self-trapped matter waves. This situation is rather different from the one reported in 2015 where three dimensional self-trapped solitons where stabilized by SOC^30–32^. Nevertheless, we found that the instability generated by SOC is completely removed when a linear potential is turned on, see Fig. 2. The first row with *γ* = 0.5 belonging to the unstable region, presents self-trapped solutions, while the second row displays the stabilizing effect of the linear potential. This suggests that the linear potential can be a very useful tool appropriate to reinforce the robustness of nonlinear waves in BECs and related fields. However, the strength of the linear potential should remain relatively small, as large values of *β* induce periodic oscillations at the top of the pseudospin densities.

### Soliton-periodic solutions

**Figure 3:**
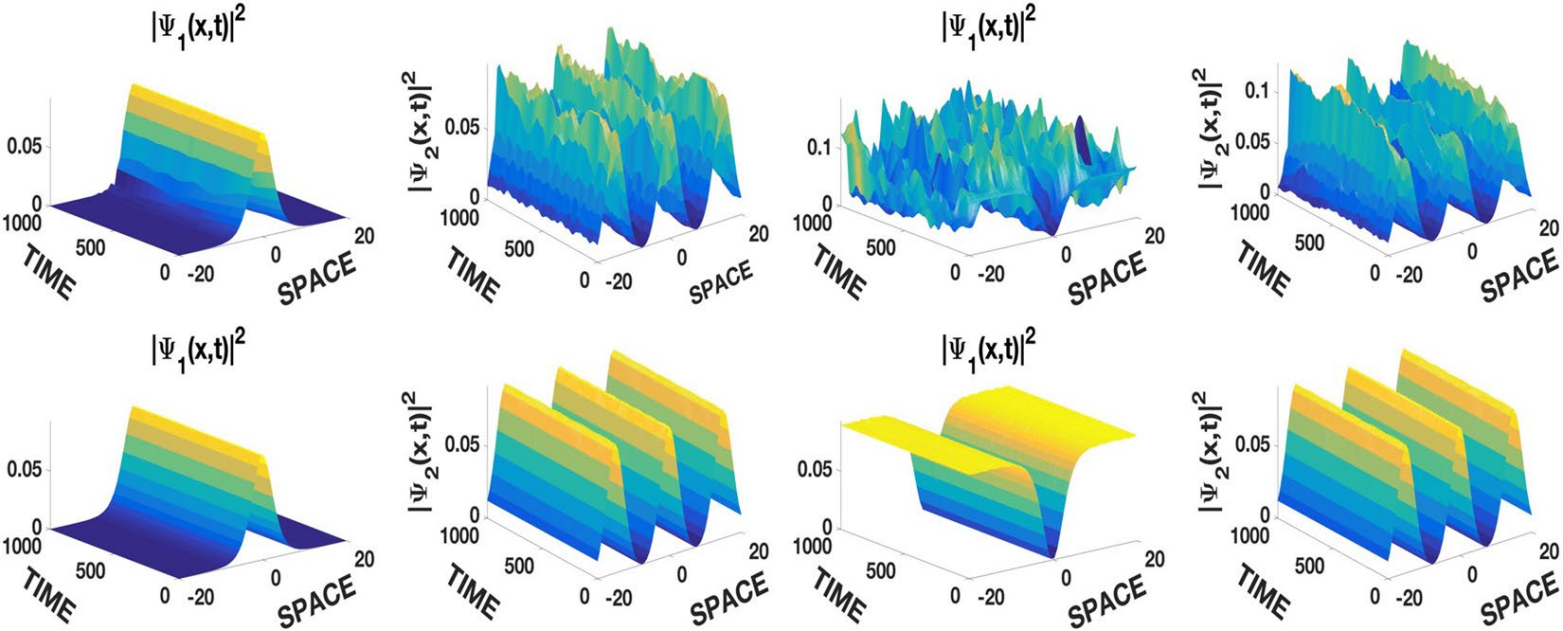
Stabilization of the unstable repulsive self-trapped solitons-periodic complexes of the first row by the linear potential in the second row. Left columns *ψ*_1,29_ and *ψ*_2,3_, right columns *ψ*_1,31_ and *ψ*_2,3_. *g*_11_ = *g*_22_ = 1 with identical conditions and parameters as in Fig. 2.

We present the coexistence of the JEF solution of type dn with bright and dark solitons in Fig. 3. Once again, the linear potential enhances the stability of the solutions that are also dynamically stable.

We would like to emphasize that all analytical solutions presented in Figs 1–3 for the case *ϕ*_1_ = *ϕ*_2_, have analogous results with respect to their robustness and regarding the effects of the SOC and that of the linear potential, during numerical simulations.

## Conclusion

In summary, we have used the F-expansion method to construct three families of solutions for spin-orbit coupled BECs with linear potential including JEFs, solitons of bright and dark types, and triangular periodic solutions. Our numerical findings show that the linear potential stabilize the solutions with large values of SOC strength while, it may be used to control important features of the center of mass like its position, velocity, and acceleration as suggested by analytical calculations. Hence, the linear potential offers the possibility to transport BECs with SOC and may even be helpful for the realization of’spin-orbit atomic lasers’. Our analytical solutions are for a wide range of physical parameters very well confirmed by extensive numerical simulations which show that the matter waves proposed in this work are robust and should be observable in experiment. An interesting future direction is the investigation of the existence and stability properties of the exotic complexes found here in higher spatial dimensions.
